# The Effect of MGCD0103 on CYP450 Isoforms Activity of Rats by Cocktail Method

**DOI:** 10.1155/2015/517295

**Published:** 2015-08-19

**Authors:** Jinzhang Cai, Qingwei Zhang, Kezhi Lin, Lufeng Hu, Yuancai Zheng

**Affiliations:** ^1^The Second Affiliated Hospital & Yuying Children's Hospital, Wenzhou Medical University, Wenzhou 325000, China; ^2^Shanghai Institute of Pharmaceutical Industry, Shanghai 200437, China; ^3^Medical Experimental Teaching Center, Wenzhou Medical University, Wenzhou 325035, China; ^4^The First Affiliated Hospital, Wenzhou Medical University, Wenzhou 325035, China

## Abstract

MGCD0103, an isotype-selective histone deacetylase inhibitor (HDACi), has been clinically evaluated for the treatment of hematologic malignancies and advanced solid tumors, alone and in combination with standard-of-care agents. In order to investigate the effects of MGCD0103 on the metabolic capacity of cytochrome P450 (CYP) enzymes, a cocktail method was employed to evaluate the activities of human CYP2B1, CYP1A2, CYP2C11, CYP2D6, CYP3A4, and CYP2C9. The rats were randomly divided into MGCD0103 group (Low, Medium, and High) and control group. The MGCD0103 group rats were given 20, 40, and 80 mg/kg (Low, Medium, and High) MGCD0103 by continuous intragastric administration for 7 days. Six probe drugs, bupropion, phenacetin, tolbutamide, metoprolol, testosterone, and omeprazole, were given to rats through intragastric administration, and the plasma concentrations were determined by UPLC-MS/MS. Statistical pharmacokinetics difference for tolbutamide in rats were observed by comparing MGCD0103 group with control group. Continuous 7-day intragastric administration of MGCD0103 slightly induces the activities of CYP2C11 of rats.

## 1. Introduction

Cytochrome P450 (CYP) enzymes are essential for most biotransformation steps of xenobiotics and endogenous molecules [1, 2]. The CYP enzymes play a critical role in drug metabolism and the interactions between supplements and drugs [3–5]. To avoid severe adverse effects from undesirable drug-drug interactions, it is highly desirable to understand the potential effects of a new chemical entity on drug-metabolizing enzymes [4, 6].

Recently, inhibition of histone deacetylases (HDACs) is recognized as a novel and validated therapeutic strategy against cancer [7, 8]. For example, SAHA and FK-228 are broad-spectrum HDAC inhibitors (HDACI) that have been approved by FDA for the treatment of refractory cutaneous T-cell lymphoma (CTCL) [9, 10]. The benzamide HDACIs, such as MS-275 and Mocetinostat (MGCD0103), selectively target HDAC 1–3 and exhibit better tolerability and efficacy in the clinical study compared with the above HDACI [11, 12]. MGCD0103 is an orally active benzamide HDACI currently being assessed in numerous phase I-II trials for hematological malignancies and solid tumors in single-agent therapy or in combination with azacitidine, gemcitabine, or docetaxel [13]. Nevertheless, many HDACIs including MGCD0103 have side effects, such as myelosuppression, fatigue, pneumonia, or cardiovascular toxicity. On the other hand, undesirable drug-drug interactions also have been reported when HDACI is coadministrated with other anticancer agents [14, 15]. Therefore, exploring the influence on CYP enzyme caused by MGCD0103 would facilitate understanding its metabolic behavior and avoid some undesirable drug-drug interactions or toxicity.

So far, no study on the effects of MGCD0103 on the metabolic capacity of CYP enzyme was reported. Therefore, in this study, six probe drugs were employed to evaluate effect of MGCD0103 on the metabolic capacity of human CYP1A2, CYP2B6, CYP2C19, CYP2C9, CYP2D6, and CYP3A4. The homology of enzymes in rat is in the order of CYP1A2, CYP2B1, CYP2C, CYP2D4, and CYP3A2 [16, 17]. The effects of MGCD0103 on rat CYP enzyme activity will be evaluated according to the changes in the pharmacokinetic parameters of six specific probe drugs.

## 2. Material and Methods

### 2.1. Chemicals

Bupropion, phenacetin, tolbutamide, metoprolol, testosterone, omeprazole (all >98%), and the internal standard diazepam (IS) were obtained from Sigma-Aldrich Company (St. Louis, USA). Ultrapure water was prepared by Millipore Milli-Q purification system (Bedford, USA). Methanol and acetonitrile (HPLC grade) were obtained from Merck Company (Darmstadt, Germany).

### 2.2. Animals

Sprague-Dawley rats (male, 220 ± 20 g) were purchased from Shanghai SLAC Laboratory Animal Co., Ltd. Animals were housed under a natural light-dark cycle conditions with controlled temperature (22°C). All forty rats were housed at Wenzhou Medical University Laboratory Animal Research Center. All experimental procedures were approved ethically by the Wenzhou Medical University Administration Committee of Experimental Animals.

### 2.3. UPLC-MS/MS Conditions

The compounds were analyzed by a UPLC-MS/MS with ACQUITY I-Class UPLC and a XEVO TQD triple quadrupole mass spectrometer that was equipped with an electrospray ionization (ESI) interface (Waters Corp., Milford, MA, USA). The UPLC system included a Sample Manager with Flow-Through Needle (SM-FTN) and a Binary Solvent Manager (BSM). Data acquisition and instrument control were performed on the MassLynx 4.1 software (Waters Corp., Milford, MA, USA).

Bupropion, phenacetin, tolbutamide, metoprolol, testosterone, omeprazole, and diazepam (IS) were separated using a Waters BEH C18 column (2.1 mm × 100 mm, 1.7 *μ*m) at constant temperature of 40°C. The initial mobile phase consisted of 0.1% formic acid and acetonitrile with gradient elution at a flow rate of 0.4 mL/min and an injection volume of 2 *μ*L. Elution was in a linear gradient, with the acetonitrile changing from 30 to 60% in 0.3–1.8 min and increasing up to 95% over 0.2 min. The acetonitrile content was maintained at 95% for 0.5 min and decreased to 30% within 0.1 min and then maintained at 30% for 0.4 min. The total run time of the analysis was 3 min.

The mass spectrometric detection was performed in a positive mode. Nitrogen was used as the cone gas (50 L/h) and desolvation gas (1000 L/h). The mass conditions were set as follows: source temperature 150°C, capillary voltage 2.5 kV, and desolvation temperature 500°C. The multiple reaction monitoring (MRM) mode with m/z 180.1 → 109.9 for phenacetin, m/z 268.1 → 115.8 for metoprolol, m/z 289.0 → 97.0 for testosterone, m/z 346.1 → 197.8 for omeprazole, m/z 271.2 → 155.1 for tolbutamide, m/z 240.1 → 184.1 for bupropion, and m/z 285.1 → 193.1 for IS was used for quantitative analysis.

### 2.4. Pharmacokinetics

Forty rats (220 ± 20 g) were randomly divided into four different dosages of MGCD0103 groups (Low group, Medium group, High group, and control group with 10 rats in each group). MGCD0103 was dissolved in corn oil as suspension at three different concentrations (20, 40, and 80 mg/mL). Three different MGCD0103 groups (Low group, Medium group, and High group) were respectively given MGCD0103 20, 40, and 80 mg/kg one time by intragastric administration at every morning and last for 7 days. Control group were given saline by same administration method. At 8 days morning, six probe drugs, bupropion, phenacetin, tolbutamide, metoprolol, testosterone, and omeprazole, were mixed in corn oil and given to the rats of three MGCD0103 groups and control group by intragastric administration at a single dosage of 10 mg/kg for bupropion, phenacetin, metoprolol, testosterone, and omeprazole and 1 mg/kg for tolbutamide.

Blood (0.3 mL) samples were collected into heparinized 1.5 mL polythene tubes from the tail vein at 0.0833, 0.5, 1, 2, 3, 4, 6, 8, 12, 24, and 48 h after intragastric administration of six probe drugs. 100 *μ*L of plasma was obtained from blood sample after centrifugation at 4000 g for 10 min. In a 1.5 mL centrifuge tube, 200 *μ*L of acetonitrile (containing 50 ng/mL IS) was added into 100 *μ*L of collected plasma sample. After vortex-mixing for 1.0 min, the sample was centrifuged at 13000 g for 15 min. Then supernatant (2 *μ*L) was injected into the UPLC-MS/MS system for analysis.

Concentration of plasma probe drugs versus time was analyzed by Version 3.0 Data Analysis System (Wenzhou Medical University, China). The main pharmacokinetic parameters of the MGCD0103 group and control group were analyzed by SPSS l8.0 statistical software.

### 2.5. Histopathology

After pharmacokinetic properties analysis, rats were deeply anesthetized with 10% chloral hydrate (i.p., 20 mg/kg). The livers of control group and MGCD0103 treated groups were rapidly isolated and immersed in freshly prepared 4% w/v formaldehyde (0.1 M phosphate buffer, pH 7.2) for 48 h and then embedded in paraffin. Then 5 *μ*m thick histologic sections were prepared and stained with routine HE method (hematoxylin and eosin). The morphological changes of liver were observed under light microscope.

## 3. Results

### 3.1. Method Validation

The concentrations of bupropion, phenacetin, tolbutamide, metoprolol, testosterone, and omeprazole in rat plasma were simultaneously determined by a sensitive and simple UPLC-MS/MS method [18]. The LLOQ for each probe drug in plasma was 2 ng/mL. The RSD of the six probe drugs was less than 15%. The calibration plot of the probe drugs is in the range of 2–2000 ng/mL (*r* > 0.995). The intraday and interday accuracy ranged from 90% to 115%. The matrix effects were more than 82% or less than 113%. The extraction recoveries were better than 85%.

### 3.2. Pharmacokinetics

**Figure 1:**
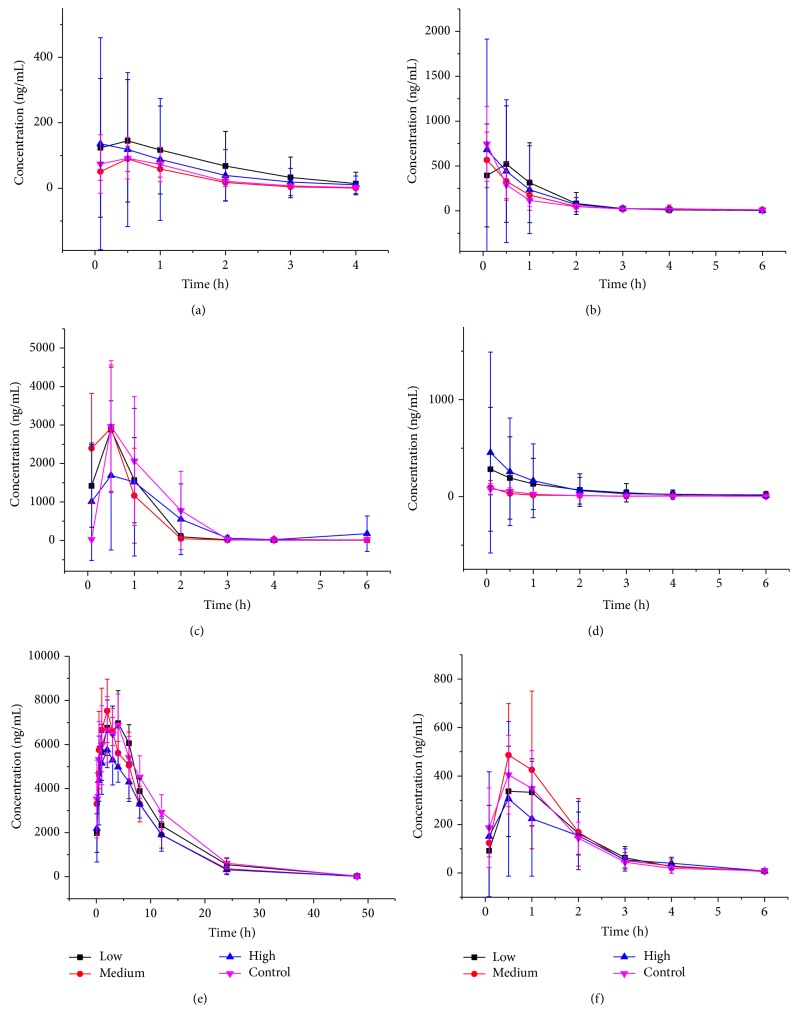
The pharmacokinetic profiles of bupropion (a), omeprazole (b), phenacetin (c), testosterone (d), tolbutamide (e), and metoprolol (f) in control group and MGCD0103 group (low, medium, and high) rats (*n* = 10). From the result, no difference in pharmacokinetic behaviors can be observed between low, medium dosage group and control group. On the other hand, no significant difference for AUC, *t*_1/2_ of omeprazole, phenacetin, metoprolol, testosterone, and bupropion (*p* > 0.05) between the high dosage group and control group was observed. However, the pharmacokinetic parameters of tolbutamide experienced obvious change with decreased AUC_(0–*t*)_ (*p* < 0.05) and increased CL (*p* < 0.05) after the dosage increase.

The main pharmacokinetic parameters of bupropion, phenacetin, tolbutamide, metoprolol, testosterone, and omeprazole calculated from noncompartment model analysis were summarized in Tables 1, 2, and 3. The representative profiles of concentration of drugs (phenacetin, metoprolol, testosterone, omeprazole, tolbutamide, and bupropion) versus time were presented in Figure 1.

From Table 3, compared with the control group, no difference in pharmacokinetic behaviors can be observed between low, medium dosage group and control group. However, the pharmacokinetic parameters of tolbutamide experienced obvious change with decreased AUC_(0–*t*)_ (*p* < 0.05) and increased CL (*p* < 0.05) after the dosage increase. This result indicates that the 7-day intragastric administration of MGCD0103 with high dosage slightly induces the metabolism of tolbutamide in rat.

On the other hand, no significant difference for AUC, *t*_1/2_ of omeprazole, phenacetin, metoprolol, testosterone, and bupropion (*p* > 0.05) between the high dosage group and control group was observed. Tables 1–3 suggested that MGCD0103 was not able to induce or inhibit the activity of CYP1A2, CYP2B1, CYP2D4, and CYP3A2 enzyme. The CL of testosterone was shortened in high dosage group, but other parameters such as AUC_(0–*t*)_, AUC_(0–*∞*)_, *t*_1/2_, *V*, and *C*_max⁡_ did not undergo changes simultaneously; this result may be attributed to experimental error.

### 3.3. Morphological Changes of Liver

**Figure 2:**
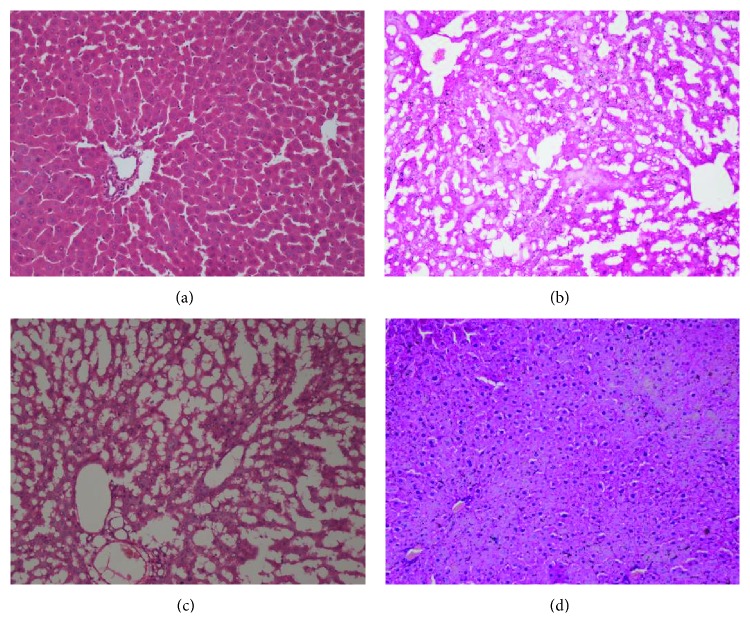
Morphological changes of liver in MGCD0103 treated group at low (a), medium (b), high (c) dosage and control group (d) (hematoxylin-eosin staining, ×200). In low dose, liver cells were arranged in funicular along with central veins and became slightly edematous (a). In middle dose, the structure of liver lobule disappeared with extensive fatty changes in the liver cells (b). In high dose, a plenty of liver cells with steatosis and small, atrophy, hyperchromatic karyopyknosis and some dark blue fragment of nucleus in lobule appeared (c).

In low dose group (Figure 2(a)), the hepatic lobule, central veins, and portal areas can be recognized at low magnification; liver cells are arranged in funicular along with central veins; the liver cells become slightly edematous. In middle dose group (Figure 2(b)), the structure of liver lobule disappeared at low magnification. The liver cells experienced extensive fatty changes; some of liver cells are pressed extensively; karyopyknosis liver cells with basophilic nucleus and eosinophilic cytoplasm appeared at high magnification. In high dose group (Figure 2(c)), the structure of liver lobule can be recognized at low magnification. A plenty of liver cells with steatosis and small, atrophy, hyperchromatic karyopyknosis with some dark blue fragment of nucleus in lobule were observed. According to the pathological changes of liver at different dosage of MGCD0103, MGCD0103 is hepatotoxic and its toxicity is dose-dependent.

## 4. Discussion

Histone deacetylases (HDACs) remove the acetyl groups from acetylated lysines of histones and also function as transcriptional corepressors [19–21]. Unlike SAHA, MGCD0103 is a nonhydroxamate isotype-selective HDACI that targets HDAC isotypes 1–3 and 11 [13]. Preclinical studies showed that MGCD0103 exhibited significant potent antitumor activity with low toxicity* in vivo*. Induction of histone acetylation in tumors by MGCD0103 has been observed to correlate with antitumor activity in mouse models with human tumor xenografts.

In general, changes in pharmacokinetics are attributed to drug-drug or drug-food interactions [22]. In pharmacokinetic interactions, drug metabolic enzymes are considered to be the most important interactive sites for drug metabolism. For example, a large number of drugs are metabolized by CYP enzymes in the liver, and more than 90% of drug-drug interactions occur at the CYP enzyme-catalyzed step [23, 24]. Similarly, supplement-drug interactions involving CYP enzyme-catalyzed metabolism are also found to cause severe adverse effect events. For these reasons, we investigated the effects of 7-day intragastric administration of MGCD0103 on the activity of CYP enzymes* in vivo*. CYP isoforms CYP1A2, CYP2D6, CYP3A4, CYP2C19, CYP2C9, and CYP2B6 were investigated in this study as more than 90% of drugs are metabolized by those 6 CYP enzymes [25, 26].

As MGCD0103 is always administrated in combination with other drugs, interactions between MGCD0103 and other drugs would increase the risk of either diminished efficacy or adverse effects. In our study, we found that 7-day intragastric administration of MGCD0103 slightly induce the metabolism of tolbutamide. This effect will result in decreased concentration of tolbutamide, which makes it hard to achieve the therapeutic effect. Tolbutamide is mainly metabolized by CYP2C11 enzyme and partly by CYP2C isoforms in the rat [27]. Rat CYP2C is homolog of human CYP2C19 and CYP2C9. CYP2C9 is involved in the oxidation of many drugs, such as losartan, torasemide, and nonsteroidal anti-inflammatory drugs, while CYP2C19 metabolizes a number of drugs, such as clopidogrel, citalopram, lansoprazole, and propranolol [28]. Therefore, the metabolism and elimination of drugs would change if they are administrated in combination with MGCD0103.

After the pharmacokinetic profiles evaluation by cocktail method, we also investigated the hepatotoxicity of MGCD by observing the pathological changes of liver after MGCD administration. The pathological changes of liver were observed at three different dosages with significant changes in high dosage and small changes in low dosage. Therefore, MGCD is hepatotoxic and its toxicity is dose-dependent. However, probe drugs were administrated to all of those rats in different groups before the pathological observation, and the probe drugs maybe cooperated with MGCD to cause hepatotoxicity. Therefore, a more systematic and comprehensive study to investigate the hepatotoxicity of MGCD will be carried out.

## 5. Conclusion

The results observed in this study would provide us with valuable information regarding the interactions of MGCD0103 with other drugs. Induction of drug metabolizing enzyme by MGCD0103 would reduce the efficacy of other drug. Additionally, concerns of hepatotoxicity of MGCD need special attention.

## Figures and Tables

**Table 1:** Pharmacokinetic parameters of bupropion and omeprazole from control group and MGCD0103 group rats (mean ± SD, *n* = 10).

Parameters	AUC_(0–*t*)_	AUC_(0–*∞*)_	*t*_1/2_	CL	*V*	*C*_max⁡_
	ng/mL∗h	ng/mL∗h	h	L/h/kg	L/kg	ng/mL
Bupropion						
Low	282.5 ± 403.2	324.2 ± 506.3	0.8 ± 0.4	128.3 ± 141.5	149.6 ± 187.0	156.7 ± 179.0
Medium	120.4 ± 45.1	122.3 ± 45.2	0.6 ± 0.2	90.6 ± 28.2	80.7 ± 52.9	98.5 ± 28.9
High	218.2 ± 460.8	235.2 ± 502.8	0.9 ± 0.5	200.6 ± 132.2	249.2 ± 205.6	156.1 ± 315.5
Control	144.8 ± 94.1	146.7 ± 95.1	0.6 ± 0.1	107.7 ± 87.6	110.2 ± 119.6	116.5 ± 93.7
Omeprazole						
Low	700.1 ± 910.2	704.9 ± 909.5	0.9 ± 0.3	29.1 ± 16.6	42.7 ± 32.0	556.4 ± 646.0
Medium	526.1 ± 251.2	539.1 ± 252.5	0.9 ± 0.6	22.3 ± 10.0	32.6 ± 24.3	610.5 ± 280.0
High	676.8 ± 1111.6	731.8 ± 1098.8	1.1 ± 0.4	31.8 ± 19.0	52.3 ± 34.7	682.5 ± 1232.1
Control	503.8 ± 199.4	528.0 ± 190.0	1.4 ± 1.0	21.2 ± 7.6	45.9 ± 38.4	745.9 ± 416.8

**Table 2:** Pharmacokinetic parameters of phenacetin and testosterone in control group and MGCD0103 group rats (mean ± SD, *n* = 10).

Parameters	AUC_(0–*t*)_	AUC_(0–*∞*)_	*t*_1/2_	CL	*V*	*C*_max⁡_
	ng/mL∗h	ng/mL∗h	h	L/h/kg	L/kg	ng/mL
Phenacetin						
Low	2973.6 ± 1807.0	2975.9 ± 1806.6	0.6 ± 0.4	6.9 ± 7.7	6.4 ± 8.2	2878.6 ± 1629.1
Medium	2910.1 ± 1758.5	2918.2 ± 1751.4	0.6 ± 0.3	4.9 ± 3.2	4.5 ± 4.6	3185.1 ± 1617.4
High	2869.8 ± 3189.9	2871.9 ± 3189.0	0.6 ± 0.2	8.5 ± 7.4	9.5 ± 10.0	2038.8 ± 1958.2
Control	3831.2 ± 2840.9	3833.6 ± 2839.3	0.5 ± 0.2	4.2 ± 3.2	3.4 ± 3.5	3151.5 ± 1872.9
Testosterone						
Low	404.5 ± 908.2	417.2 ± 927.8	1.7 ± 0.8	104.8 ± 49.0	259.1 ± 168.6	302.1 ± 630.9
Medium	81.3 ± 28.8	106.5 ± 45.1	2.2 ± 2.4	109.0 ± 43.8	253.2 ± 204.3	93.2 ± 75.3
High	490.7 ± 1044.2	524.5 ± 1042.4	4.0 ± 7.1	64.9 ± 33.4^∗^	254.0 ± 291.0	461.8 ± 1032.1
Control	85.9 ± 40.6	89.9 ± 43.9	1.0 ± 0.6	143.8 ± 89.6	161.1 ± 82.2	88.1 ± 54.2

MGCD0103 group was compared with the control group, ^∗^ *p* < 0.05.

**Table 3:** Pharmacokinetic parameters of tolbutamide and metoprolol in control group and MGCD0103 group rats (mean ± SD, *n* = 10).

Parameters	AUC_(0–*t*)_	AUC_(0–*∞*)_	*t*_1/2_	CL	*V*	*C*_max⁡_
	ng/mL∗h	ng/mL∗h	h	L/h/kg	L/kg	ng/mL
Tolbutamide						
Low	82724.8 ± 13265.9	83176.9 ± 13264.2	5.2 ± 1.2	0.012 ± 0.002	0.090 ± 0.014	7939.9 ± 1199.1
Medium	72448.6 ± 14850.4	72761.4 ± 14754.7	4.7 ± 1.0	0.014 ± 0.003	0.093 ± 0.010	8038.6 ± 1318.2
High	64052.9 ± 15988.6^∗^	64353.2 ± 15998.9^∗^	4.4 ± 1.2	0.016 ± 0.003^∗^	0.099 ± 0.020	6208.9 ± 1115.5
Control	88701.2 ± 19274.8	89852.0 ± 19168.1	5.3 ± 0.7	0.012 ± 0.004	0.088 ± 0.019	7343.2 ± 1447.5
Metoprolol						
Low	698.1 ± 348.6	703.8 ± 351.6	0.8 ± 0.1	18.2 ± 10.5	21.6 ± 13.5	370.7 ± 161.1
Medium	836.1 ± 499.8	843.7 ± 498.4	0.7 ± 0.3	14.1 ± 4.8	15.7 ± 9.1	520.9 ± 300.9
High	1031.8 ± 1261.2	1046.4 ± 1258.4	1.2 ± 0.4	22.9 ± 17.4	43.6 ± 39.6	1185.3 ± 2379.0
Control	709.2 ± 255.8	724.2 ± 255.8	0.8 ± 0.2	15.8 ± 6.7	18.0 ± 8.6	426.1 ± 159.3

MGCD0103 group was compared with the control group, ^∗^ *p* < 0.05.
